# Total hip arthroplasty in osteonecrosis secondary to sickle cell disease

**DOI:** 10.1007/s00264-018-4001-0

**Published:** 2018-06-15

**Authors:** Mohamed Zubair Farook, Moji Awogbade, Karthik Somasundaram, Ines L. H. Reichert, Patrick L. S. Li

**Affiliations:** 1 Department of Trauma & Orthopaedics, King’s College Hospital NHS Foundation Trust, King‘s College London, Denmark Hill, London, SE5 9RS UK; 2 Department of Haematological Medicine, Kings College Hospital NHS Foundation Trust, Denmark Hill, London, SE5 9RS UK; 3 Morriston Hospital, Swansea, Wales UK

**Keywords:** Sickle cell disease, Osteonecrosis, THA, Revision

## Abstract

**Background:**

Sickle cell disease (SCD) is a multisystem disease, and the predominant articular manifestation is osteonecrosis (ON). Total hip arthroplasty (THA) is technically challenging, and the complication rates are high. In this retrospective study, we have analysed the outcome of THA in a cohort of patients with SCD at our institution.

**Materials and methods:**

We identified 34 THAs between 1999 and 2016 in 30 patients (mean age 37 years) from our SCD database. Co-morbidities, both sickle and non-sickle-related, were documented. Complications and indications for revision surgery were analysed.

**Results:**

An uncemented prosthesis was predominantly used. The mean follow-up was 10.5 years (range 1–18). Six patients had revision surgery (17.6%), 2 (5.8%) for Prosthetic Joint Infection (PJI), and 4 (11.7%) for osteolysis of the acetabular component.

**Conclusion:**

Our revision rates were comparable to the published literature. Our combined sickle cell clinic and the coordinated multidisciplinary management have been successful in reducing morbidity.

## Introduction

Sickle cell disease (SCD) is a multisystem disease affecting the brain, kidneys, lungs, bones and cardio vascular systems. Haemoglobin polymerization occurs due to the inheritance of two abnormal beta globin genes [9]. This leads to erythrocyte rigidity and vaso-occlusion, which is central to the pathophysiology of this disease [22]. Recurrent episodes of vaso-occlusion and inflammation result in progressive damage to organs including bone. The predominant articular manifestation of SCD is osteonecrosis (ON), invariably affecting the femoral head in 50% of patients [9, 25]. Multidisciplinary management including drug therapy, stem cell transplantation and advances in total hip arthroplasty (THA) has significantly improved the quality of life of patients. Only 50% of patients with sickle cell anaemia survive beyond the fifth decade [22]. However, THA is technically very challenging and the complication rates are high when compared with other indications, including other causes of ON. Intra-operative complications such as femoral perforation and fractures are not infrequent. Failure rates of up to 62.5% have been reported [12]. In this retrospective study, we have analysed the outcome of THA in a cohort of patients with sickle cell disease from our institution, which has a catchment area with a significant afro-Caribbean population.

## Materials and methods

### Data collection

We identified 30 patients with 34 THAs performed between 1999 and 2016 from our SCD database. Two patients died from medical complications of sickle cell disease, one of them ten years and other one two years post-operatively. Patients were reviewed in a combined Sickle Cell Clinic pre-operatively, postoperatively at six weeks, and then annually by a consultant haematologist (MA) and the senior author (PLSL).

Case notes, Electronic Patient Record (EPR), and Picture Archive and Communication System (PACS) were used for data collection. Co-morbidities, both sickle and non-sickle related, were documented. The Oxford Hip Score (OHS) was as per routine recorded, but not included, as no scoring system, including the OHS, is validated for SCD due to the fact that these patients have residual pain from other sources [19]. Radiographs were reviewed by two independent observers. Pre-operative radiographs were analysed for staging of ON using the Steinberg Classification [23] and also for femoral canal medullary sclerosis. Post-operative radiographs were reviewed for component loosening using the Charnley [7] and Gruen [10] methods for cemented and Engh [8] method for uncemented prostheses. Heterotopic ossification (HO) was assessed according to Brookers [5] criteria. Intra-operative and post-operative, medical as well as surgical, complications were reviewed. Indications for revision surgery were analysed.

### Peri-operative care

All patients received pre-operative exchange transfusion aimed at optimizing the haemoglobin (Hb) > 10 g/dL, and Hb S concentration < 30% was the usual standard of care. A combination of spinal anaesthesia and either sedation or general anaesthesia was routinely used. Normothermia was maintained intra-operatively using forced-air patient warming system (Bair hugger). Patients were kept well hydrated, and the urine output was monitored. Broad-spectrum prophylactic antibiotics were administered at induction. During the last five years, the protocol also included 1 g of Tranexamic acid. Intravenous antibiotics were continued postoperatively for 48 hours.

### Surgical technique

Surgery was performed using Freeman’s transgluteal approach [24]. In patients with a sclerotic canal, femoral preparation was done meticulously using a guidewire to locate the canal before sequential intramedullary reaming was undertaken. During the earlier period of study (1999–2004), hybrid or cemented Exeter™ (Stryker Howmedica) system was predominantly used. Uncemented JRI prosthesis was used in one patient bilaterally. In the later period, uncemented Corail-Pinnacle (Depuy), S-ROM (Depuy), or ABG (Stryker) prosthesis was used.

### Post-operative care

Patients were managed post-operatively by the haematology, orthopaedic and pain teams. The patients were allowed to weight-bear according to comfort. In cases where femoral perforation occurred, it was managed by bypassing the perforation adequately with the femoral stem and patients were restricted to touch-weight bearing for six weeks. Mechanical thromboprophylaxis consisted of intermittent pneumatic compression pumps until the patients started mobilizing out of bed and anti embolism stockings for six weeks. Low molecular weight heparin was given as chemical prophylaxis for four weeks post-operatively.

## Results

The mean age at surgery was 36.7 years (range 20–59). There were 12 men and 18 women. Four patients had staged bilateral THA. Twenty-four patients (77%) were homozygous to sickle cell gene (Hb SS), four were Hb SC, one had HbSß and one had HbSS G Philadelphia variant. The majority of patients were stage 4 or above according to the Steinberg [23] classification. The associated co-morbid conditions are illustrated in Table 1. Invariably, all patients had more than one admission for an acute sickle complication at some point unrelated to the THA episode. This includes 26.6% who had admissions for acute chest syndrome. In 31% of patients, the disease affected other joints mainly around the shoulder and knee.

**Table 1:** Co-morbidities

Co-morbidities	Patients (*n*)
Acute chest syndrome Cerebrovascular accident Renal disease Pulmonary embolism Sickle retinopathy Priapism Transfusional iron overload	8 4 5 3 6 2 6

There were 26 uncemented, five hybrid and three cemented THAs. Stryker ABG (*n* = 3), Depuy S-ROM (*n* = 4), JRI (*n* = 2) and Depuy Corail-Pinnacle (*n* = 17) were the different uncemented prostheses used. Exeter™ (Stryker Howmedica) was used for hybrid and cemented hips. Since 2005, most of the THAs, except one, were performed uncemented. Screws were used to augment acetabular fixation in about 38.7% (12 cases) of hybrid and uncemented THAs. In the uncemented group, ceramic on ceramic (CoC) was the most commonly used bearing couple in 21 cases. Metal on metal (MoM) and metal on polyethylene (MoP) were used in two cases each respectively. Ceramic on polyethylene (CoP) was used in one case.

**Fig. 1:**
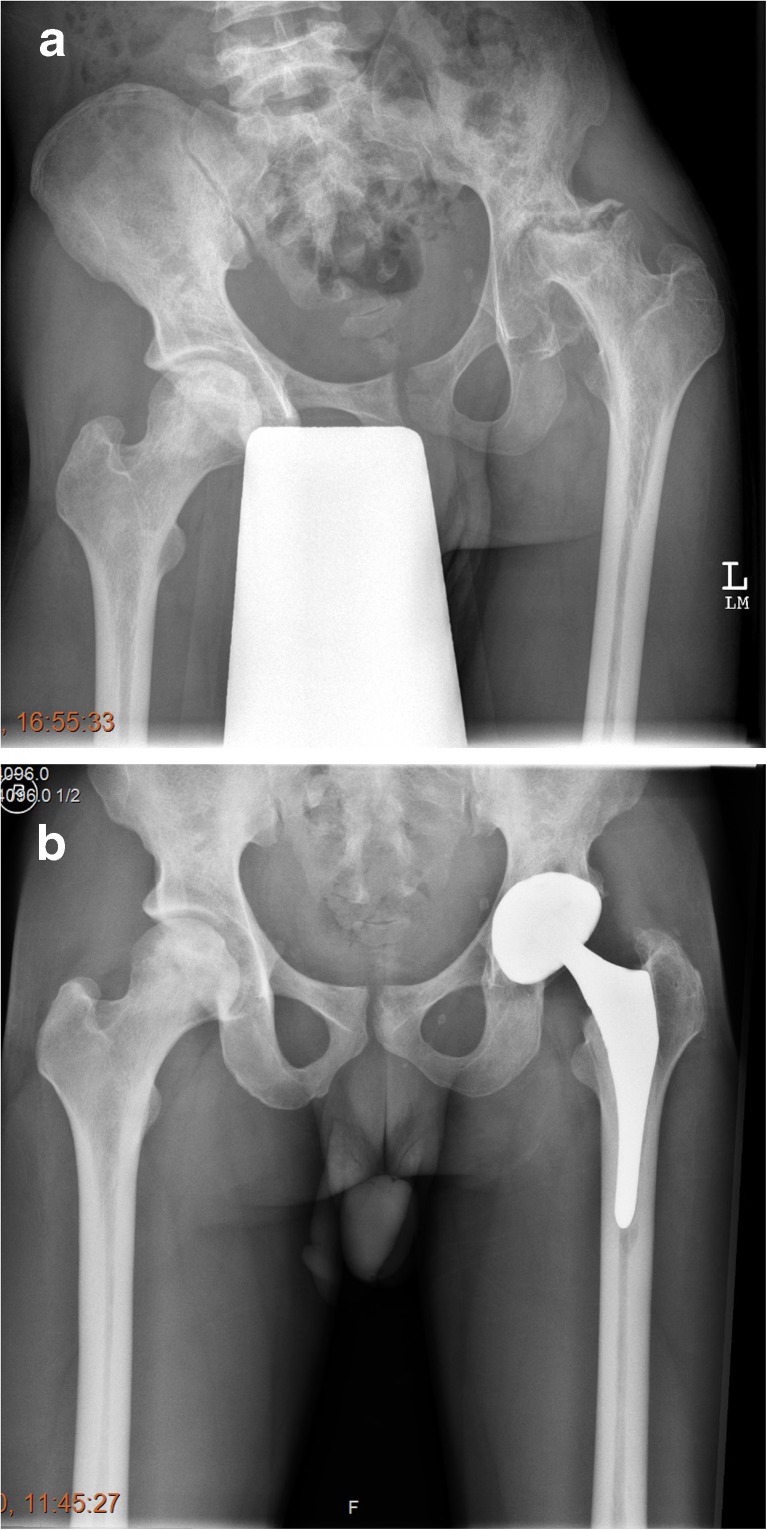
**a** Steinberg Stage VI Osteonecrosis left hip with severe femoral canal Stenosis and stage III right hip. **b** Radiographs 8 years postoperatively showing well-integrated Stryker ABG uncemented components

**Fig. 2:**
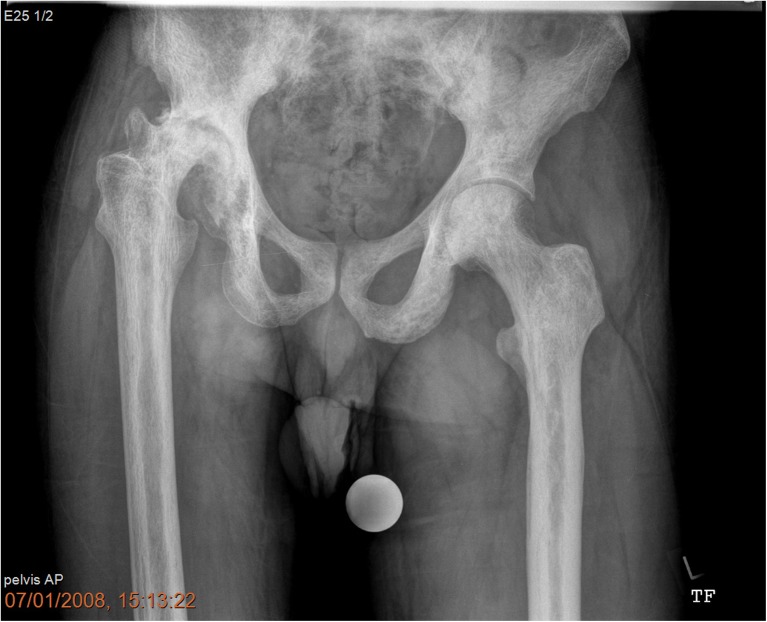
Pre-operative radiographs of a 48-year-old male, with Steinberg stage VI osteonecrosis right hip with “femur within femur” appearance

The femoral canal was sclerotic needing sequential cannulated intramedullary reaming in 24 cases (70.5%). An example is illustrated in Figs. 1a, b and 2. Figure 2 also illustrates “ Femur within femur” appearance. The mean follow-up was 10.5 years (range 1–18). Heterotopic ossification was observed in 23.5% (8 cases), mostly Brookers [5] grade 1 or 2. One patient had pulmonary embolism. There were two cases of femoral shaft perforation. There were no dislocations or nerve injury.

Six patients required revision surgery (17.6%), four in the uncemented and two in the hybrid group. Two (5.8%) of these were for prosthetic joint infection (PJI). Both were in the uncemented group. One of the patients who had an uncemented Corail-Pinnacle CoC THA was diagnosed with deep infection two years post-operatively while he was abroad and had a single stage fully cemented THA. The second patient in this group had an uncemented ABG CoC THA and developed infection one year post-operatively and underwent a two-stage revision THA with Depuy S-ROM system. Revision surgery was performed in four (11.7%) patients for polyethylene wear and osteolysis of acetabular component. One patient with bilateral uncemented THAs had acetabular revision for both hips at 11 and 12 years post-operatively. The other two patients had hybrid Exeter THAs and required revision of the acetabular component at 12 and 13 years respectively. One amongst them had a periprosthetic acetabular fracture secondary to osteolysis. He had significant end-stage renal failure, on dialysis and was at high risk for complex revision. We waited until the fracture healed and then operated using extensive debridement, bone grafting, retained the shell, cemented a polyethylene liner and performed cement in cement Exeter stem revision.

There were no cases of stem loosening. Apart from the initial two hips, we have not noticed any aseptic loosening or symptomatic polyethylene wear in our uncemented THAs. Out of the surviving 22 uncemented hips, 20 stems were Engh [8] type 1 (stable bony ingrowth) and two of them type 2 (stable fibrous ingrowth).

**Fig. 3:**
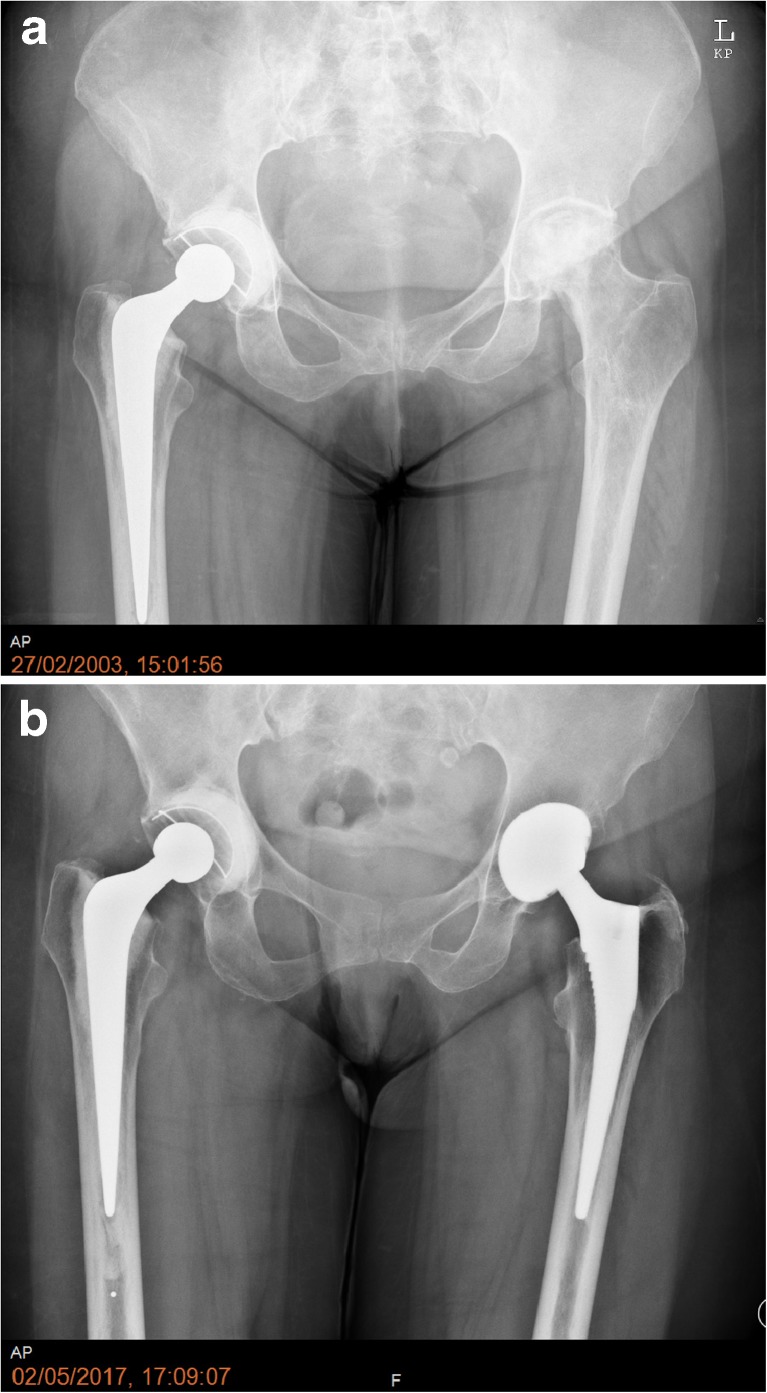
**a** One-year post-operative radiographs of a 45-year-old female with a cemented Exeter THA and pre-operative left side with Steinberg stage 5 ON. **b** Fifteen-year post-operative radiographs right side with minimal eccentric polyethylene wear and 14-year post-operative left uncemented THA

Amongst the three cemented THAs, two had minimal eccentric polyethylene wear, but doing well clinically at 13 and 15 years post-operatively (Fig. 3a, b). One patient developed aseptic loosening and migration of the acetabular component at nine years post-operatively. Due to severe co-morbidities, this patient was not fit for revision surgery. Two hybrid THAs 13 and 16 years post-operatively also had asymptomatic eccentric polyethylene wear. The remainder of the patients who had primary THAs and patients who had revision surgery were doing well clinically and radiologically at the latest follow-up.

## Discussion

A report of the national sickle cell surgery study group [25] has observed an overall serious peri-operative complication rate of up to 67%. A nationwide study by Perfetti et al. [21] concluded that patients with SCD admitted for arthroplasty had a longer and more complicated length of stay compared to other patients. Our average length of stay was 10.7 days. Infection and aseptic loosening are significant concerns in this group of relatively young patients. Our deep infection rate was 5.8%, and aseptic loosening was 11.7%.

Rates of dislocation in the literature vary between 0.75 and 3.7% [3, 15, 17–19]. We did not have any dislocation in our series. We also did not have any nerve injuries.

We encountered difficulty with the femoral canal in two thirds of the cases. We observed “femur within femur” appearance in a few cases, which is a thin cortical lining inside the true cortex as described by Ilyas et al. [17]. However, we did not have to manufacture an anterior femoral cortical window to prepare the femoral canal as described by Hug et al. [16]. We had only two cases (5.8%) of intra-operative femoral shaft perforation. Rates of femoral perforation and periprosthetic fractures range from 1.9 to 14.2% in the literature [1, 4, 15, 17, 19].

So far, the largest series published on THA in SCD is by Hernigou et al. [15], 312 THAs in 244 patients with a mean follow-up of 13 years. The rates of PJI, aseptic loosening and revision rates were 3, 8 and 13.5% respectively. The ten year survival rate was 89%. Interestingly, all their hips were cemented in contrast to other studies which showed higher failure rate with cemented THA [1, 2, 6]. They attribute their success to the use of “French Paradox” technique [20]. This involves using a largest possible rectangular canal filling titanium alloy stem without trying to obtain a continuous cement mantle. They feel that the rectangular cross section and direct load transfer to the bone by close cortical contact provided intrinsic stability within the femur which in turn might have protected the cement mantle. Hernigou has also published extensively about the natural history of both symptomatic and asymptomatic osteonecrosis in adults with sickle cell disease [13, 14].

Bankes et al. [19] reviewed their results of 52 cementless THAs, with a mean follow-up of five  years. They have not observed any case of aseptic loosening and only one case of infection post revision for dislocation. Mont et al. [18] favour an uncemented prosthesis for sickle cell ON, with only a 5% revision rate for aseptic loosening. They did not observe any significant difference compared to a cohort of THAs performed for ON related to other causes. Gulati et al. [11] observed a mean follow-up of 3.8 years in 50 THAs and have not encountered any cases of infection, dislocation or aseptic loosening.

Good numbers of studies have recently been published from the Middle East. Azam et al. [4] studied 87 uncemented THAs and showed a survival rate of 92.6% in 7.5 years. Ilyas et al. [17] have reported long term results of uncemented THAs in 133 hips with a mean follow-up of 14.6 years. They observed 94.1% survival at 15 years. The deep infection rate was 3.76%. AlOmran [3] has compared the results of cemented and uncemented THAs in his study. In a group of 136 THAs, 46 were cemented and 90 were uncemented. The failure rate was 61% in the cemented group compared to 22.3% in the uncemented group. Results from the literature are summarized in Tables 2, 3 and 4.

**Table 2:** Literature—cemented THA

Author	No. of hips	Mean follow-up (years)	PJI rates (%)	Aseptic failure acetabulum (%)	Aseptic failure femur (%)	Revision rate (%)
Al Mousawi et al. [2] 2002	37	9.5	2	20		20
Hernigou et al. [15] 2008	312	13	3	8	5	13.5
AlOmran [3] 2010	46	12	2.2	36.9	21.7	60.8

**Table 3:** Literature—uncemented THA

Author	No. of hips	Mean follow-up (years)	PJI rates (%)	Aseptic failure acetabulum (%)	Aseptic failure femur (%)	Revision rate (%)
AlOmran [3] 2010	90	5	1.1	21.1		22.2
Issa et al. [18] 2013	42	7.5	4.7	5	5	11.9
Bankes et al. [19] 2015	52	5.1	1 case post revision	0	0	3.8
Gulati et al. [11] 2015	50	3.8	0	0	0	0
Azam et al. [4] 2016	84	7.5	3.5	8.3		9.5
Ilyas et al. [17] 2018	133	14.5	3.76	0.75	0.75	8.2

**Table 4:** Literature—studies with a mix of uncemented, cemented and hybrid

Author	No. of hips	Mean follow-up (years)	PJI rates (%)	Aseptic failure acetabulum (%)	Aseptic failure femur (%)	Revision rates (%)
Acurio et al. [1] 1992	35	8.6	20	46	46	40 (Cem 59/uncem 22)
Clarke et al. [6] 1987	27	5.5	3	N/A	N/A	59
Hanker et al. [12] 1988	16	6.5	2	25	25	63
This study	34	10.5	5.8	11.7	0	17.6

*N/A* not available

## Conclusion

Our study has some limitations in that it is retrospective with small numbers especially in the cemented and hybrid group. We found these cases technically very challenging. Especially, the femoral canal frequently needed to be reamed with sequential cannulated intramedullary reamers. Our revision rates were comparable to the published literature, and most of them were either in the hybrid or earlier uncemented prosthesis. Our combined sickle cell clinic as well as the coordinated multidisciplinary inpatient management of these patients has been successful in reducing both medical and surgical morbidity.

Our current preferred implant of choice is uncemented THA with ceramic on ceramic bearings. We plan to continue this study in the future with a greater number of patients and also with extended follow-up, and this will hopefully help to provide further answers in this challenging cohort.
